# A novel risk model consisting of nine platelet-related gene signatures for predicting prognosis, immune features and drug sensitivity in glioma

**DOI:** 10.1186/s41065-024-00355-7

**Published:** 2024-12-20

**Authors:** Sanlin Wei, Junke Zhou, Bin Dong

**Affiliations:** 1 Dalian Medical University, Dalian, Liaoning Province 116000 China; 2 Department of Neurosurgery, the First Affiliated Hospital of Dalian Medical University, Dalian, Liaoning Province 116000 China; 3 Department of Nephrology, the First Affiliated Hospital of Dalian Medical University, Dalian, Liaoning Province 116000 China

**Keywords:** Glioma, Platelet, Prognostic signature, Immune infiltration, Molecular docking

## Abstract

**Background:**

Glioma is a malignancy with challenging clinical treatment and poor prognosis. Platelets are closely associated with tumor growth, propagation, invasion, and angiogenesis. However, the role of platelet-related genes in glioma treatment and prognosis remains unclear.

**Results:**

A prognostic risk model was established using nine platelet-related prognostic signature genes (*CAPG*,* CLIC1*,* GLB1*,* GNG12*,* KIF20A*,* PDIA4*,* SULF2*,* TAGLN2*, and *WEE1*), and the risk score of samples were calculated. Subsequently, the glioma samples were divided into high- and low-risk groups based on the median values of risk scores. scRNA-seq analysis revealed that the prognostic genes were primarily located in astrocytes and natural killer cells. The immune infiltration proportions of most immune cells differed significantly between high- and low-risk groups. Moreover, we found AZD7762 as a potential candidate for glioma treatment.

**Conclusion:**

Nine platelet-related prognostic genes identified as prognostic signatures for glioma were closely associated with the TME and may aid in directing the clinical treatment and prognosis of gliomas.

**Supplementary Information:**

The online version contains supplementary material available at 10.1186/s41065-024-00355-7.

## Highlights

Nine platelet-related prognostic signature genes were identified to establish a risk score and divide patients with glioma into high- and low-risk groups.

The risk score was an independent prognostic model, and a nomogram was constructed to verify the model.

Platelet-related prognostic signature genes could be used as targets for glioma therapy.

Tumor microenvironment was closely related to glioma treatment.

The drug AZD7762 is a potential candidate for glioma treatment.

**Supplementary Information:**

The online version contains supplementary material available at 10.1186/s41065-024-00355-7.

## Background

Gliomas are the most common primary cancer of the brain and account for approximately 44.6% of intracranial tumors [1, 2]. They share features of normal glial cells [3], and patients with gliomas often have limb dysfunction that seriously affects their quality of life. Glioblastoma (GBM) is among the most aggressive high-grade gliomas with a short survival time [4, 5]. Gliomas are generally derived from glial or precursor cells; their lesions are invasive and affect the surrounding tissue cells [6]. In addition, the molecular mechanism of gliomas is complex, resulting in ineffective clinical treatments and poor prognosis [7]. While medical treatments, including surgery, radiation, and chemotherapy, can reduce the risk of recurrence in gliomas, they result in an unsatisfactory overall survival rate because of the easy infiltration and diffusion, easy postoperative recurrence, and unfavorable prognosis of gliomas [8–11]. Therefore, it is critical to develop novel prognostic models and identify effective targeted drugs for patients with glioma.

Platelets, the smallest components of circulating blood cells, not only promote hemostasis and coagulation but also influence the growth, metastasis, and recurrence of tumors, thereby affecting patient prognosis [12–15]. Platelets and their growth factors can promote tumor metastasis by affecting tumor neovascularization [16], promoting tumor growth [17], and helping tumor cells evade natural killer (NK) cell recognition [18]. In turn, tumor cells can induce platelet activation and aggregation [18]. The interaction between cancer cells and platelets can regulate the malignant progression of tumors. This interaction may arise from the unique anatomical characteristics of gliomas—solid tumors with abundant blood vessels. Therefore, investigating the potential function of platelets in gliomas is of utmost importance. Glioma-associated cells may enhance immunosuppression in the glioma microenvironment through platelet regulation, thereby promoting glioma growth, aggressiveness, and neovascularization [19]. Activated platelets regulate the immune response in gliomas by inhibiting the migration of regulatory T cells (Tregs) [20]. Current studies on platelets in glioma have mainly focused on the prognostic value of the platelet-to-lymphocyte ratio and platelet counts [21–24]. Additionally, previous studies have stated that CD276 (B7-H3), GATA3, and galectin-3 enable prognosis prediction in glioblastoma [25], and that correlation between lower balance of Th2 helper T-cells and lower expression of PD-L1/PD-1 axis genes also estimate prognosis in glioblastoma [26]. However, the association between platelet-related genes (PRGs) and glioma prognosis remains unclear.

PRGs reportedly exhibit favorable prognostic guidance effects in other types of tumors, such as pancreatic cancer [14, 27], lung squamous cell carcinoma [28], triple-negative breast cancer [12], esophageal cancer [29] hepatocellular carcinoma [30]. These findings demonstrated the feasibility of establishing a prognostic model using PRGs for gliomas. In this study, we aimed to establish a prognostic risk model for immunotherapy in patients with glioma and describe the role of PRGs in the prognosis and tumor microenvironment (TME) of gliomas.

First, we performed a well-rounded analysis of genes available in public glioma datasets and identified platelet-related prognostic signature genes for gliomas using various bioinformatic tools. The expression of prognostic signature genes in the immune cells was also validated. We then established a platelet-related prognostic risk model based on these genes and verified it using multiple datasets. Subsequently, the tumor immune microenvironment and genetic mutations were analyzed, and the connection between the prognostic model and immune cells was determined. Finally, the active drug acting on the prognostic signature genes was identified. Our study can provide a novel role for glioma prognosis and inform personalized treatment for gliomas.

## Methods

### Data source

The profiles and clinical trait data of The Cancer Genome Atlas (TCGA) Glioblastoma Multiforme, TCGA Low Grade Glioma, and normal samples for Genotype-Tissue Expression were downloaded from the website of University of California Santa Cruz. After excluding samples with incomplete survival and clinical information, 1,141 normal cases and 694 glioma samples were obtained. This TCGA cohort then served as a training set, whereas the Chinese Glioma Genome Atlas (CGGA) mRNAseq_325 and CGGA mRNAseq_693 datasets downloaded from the CGGA [31] database served as the validation sets. The single-cell RNA sequencing (scRNA-seq) data GSE138794 was obtained from the Gene Expression Omnibus [32] database.

We used “platelet” as the keyword to search the public databases, including the Molecular Signatures Database [33], AmiGo 2 (https://amigo.soybase.org/amigo/amigo/landing) database, and the GeneCards human gene database [34]. After filtered with the standards of protein coding and relevance score > 1, 480, 367, and 4028 PRGs were respectively obtained. Additionally, 547 PRGs were obtained from the published literature [35]. After removing duplicate genes, a total of 4367 PRGs were included, and used for subsequent analysis in this study (Supplementary Table 1).

### Identification and enrichment analysis of characteristic genes

The R package “DESeq2” [36] was used to identify differentially expressed genes (DEGs) on 694 glioma and 1,141 normal samples according to adjusted *p*-values (p-adj) < 0.05 and |log_2_ fold change (FC)| > 1. Differentially expressed platelet-related genes (DEPRGs) were identified using overlapping DEGs and PRGs for further analysis. Characteristic genes related to survival status (*p* < 0.05) were identified using univariate Cox regression analysis of DEPRGs. We performed Gene set enrichment analysis (GSEA), Gene Ontology (GO) analysis, and the Kyoto Encyclopedia of Genes and Genomes (KEGG) analysis to explored the function and pathways of characteristic genes using the R package “clusterProfiler” with p-adj < 0.05 considered statistically enriched. “H.all.v2023.2.Hs.entrez.gmt” was set as a reference set for the GSEA analysis.

### Development and validation of the platelet-associated prognostic model

Based on the characteristic genes, the LASSO regression analysis was performed using the R package “glmnet” with a random seed of three to further shrink the platelet-related prognostic signature genes and construct the most suitable prognostic risk model. Subsequently, the prognostic model genes were confirmed, and the risk scores were imputed as follows:

$$\:risk\:score\:=\:\sum\:_{i=1}^{n}{\beta\:}_{i}\:\times\:\:{Exp}_{i}$$

where *β* indicates the LASSO regression coefficient, *i* indicates nine platelet-related prognostic genes, and the *Exp* indicates the expression. In TCGA cohort, glioma samples were categorized into high- or low-risk groups according to the median risk score. The survival curves were established using the R packages “survminer” and “survival” to determine the differences in survival between the two groups. To determine the reliability of the prognostic model, the R package “timeROC” was utilized to plot the receiver operating characteristic (ROC) curves of the patients [37]. In addition, the CGGA mRNAseq 325 and CGGA mRNAseq 693 datasets were used to verify the properties of the prognostic model for accurately predicting glioma.

### Nomogram construction

The risk scores and clinical parameters (age and gender) of patients with glioma were evaluated using univariate Cox regression analysis. Multivariate Cox regression analysis was performed to confirm whether the risk score was an independent predictive factor for glioma outcomes. Based on the independent predictor, a nomogram was established to forecast the survival probability of glioma patients at 1-, 2-, and 3-year using the R package “rms” in TCGA. A calibration diagram was used to evaluate the accuracy of the nomogram.

### Tumor microenvironment analysis

The infiltration proportions of 22 immune cells in glioma samples was appraised using the “CIBERSORT” [38]. Correlations among the prognostic signature genes, 22 immune cells, and risk scores were explored, and the infiltration proportions of 28 immune cells in the TME was determined using the “ssGSEA” analysis. ESTIMATE [39] was used to calculate the immune, stromal, and estimate scores of the TME. In particular, the association between the risk score and immune checkpoint genes [40] was calculated to investigate the connection between the prognostic model and immunotherapy.

### Immunohistochemistry data analysis

The protein expression data for these prognostic model genes were downloaded from the Human Protein Atlas database [41]. Immunohistochemistry (IHC) was used to estimate the protein levels of prognostic signature genes in the glioma and normal tissues.

### scRNA-seq data analysis

The R package “Seurat” was used to perform scRNA-seq analysis [42]. The following quality measures were adhered to: removal of three or fewer cells, removal of low-quality cells with fewer than 200 genes, and exclusion of 5% mitochondrial genes. The remaining cells were normalized using the R package “NormalizeData” function. Principal component analysis was performed on single-cell samples, and the top 20 samples was visualized using a uniform manifold approximation and projection algorithm. The R packages “singleR” and “celldex” were used to identify and annotate different cell clusters.

### Genetic mutation and candidate drugs susceptibility data analysis

Mutation differences between high- and low-risk groups in TCGA cohort was analyzed using the R package “maftools” and visualized with waterfall maps. Based on two pharmacogenomic databases (Cancer Therapeutics Response Portal [CTRP] and Genomics of Drug Sensitivity in Cancer [GDSC]), the drug sensitivity was determined by counting the half-maximal inhibitory concentration (IC_50_) of the drug candidates using the R package “oncopredict” (*p* < 0.05). Finally, the drugs that were significantly different between the two risk groups and had lower IC_50_ in the high-risk group were selected. Additionally, the ADMETlab 3.0 online platform was used to predict the clinical effects and possible side effects of drug candidates.

### Statistical analysis

The R software (version 4.4.0) was used for statistical analysis. Survival curves were generated using Kaplan–Meier (KM) curves. Correlation analysis was performed using the cor function, and the Spearman’s rank correlation coefficient was used as the statistical method. Between-group individual differences were evaluated using the Wilcoxon signed-rank test. Statistical significance was set at *p* < 0.05.

## Results

### Research schematic diagram

Supplementary Fig. 1 illustrates the research process.

### Identification of DEPRGs

**Fig. 1:**
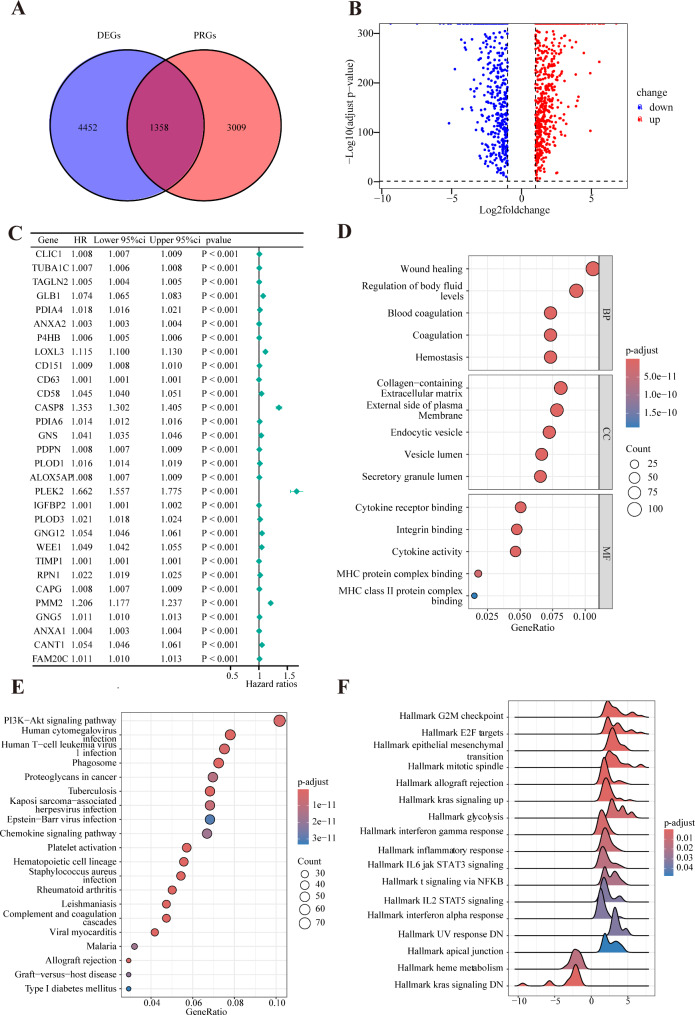
Identification and functional enrichment analysis of characteristic genes. (**A**) Venn plots showing 1,358 common genes in DEGs and PRGs. (**B**) Forest plots showing the results of the univariate Cox analysis for the top 30 genes. (**C**) Volcano plot of 1,030 characteristic genes in glioma samples compared to those in normal samples. Enrichment analysis of GO (**D**), KEGG (**E**), and GSEA (**F**) based on 1,030 characteristic genes. DEGs, differentially expressed genes; PRGs, platelet-related genes; GSEA, Gene set enrichment analysis; GO, Gene Ontology; KEGG, Kyoto Encyclopedia of Genes and Genomes

In total, 5810 DEGs and 4367 PRGs were identified, and 1358 DEPRGs were identified by overlapping DEGs and PRGs (Fig. 1A). Univariate Cox regression analysis identified 1030 characteristic genes associated with overall survival in patients with glioma (Fig. 1B), and top30 characteristic genes associated with overall survival in patients with glioma were displayed (Fig. 1C). Enrichment analysis was performed to better analyze the biological functions of the identified 1030 characteristic genes. GO analysis revealed that the characteristic genes were significantly enriched in “wound healing” (*p* = 9.84 × 10^− 43^), “regulation of body fluid levels” (*p* = 1.61 × 10^− 38^) and “coagulation” (*p* = 8.82 × 10^− 38^) of biological process; and “collagen-containing extracellular matrix” (*p* = 2.84 × 10^− 26^), “external side of plasma” (*p* = 6.61 × 10^− 26^), and “endocytic vesicle” (*p* = 5.98 × 10^− 26^) of cellular component; as well as “cytokine receptor binding” (*p* = 8.06 × 10^− 15^), “integrin binding” (*p* = 1.83 × 10^− 16^), and “cytokine activity” (*p* = 1.07 × 10^− 13^) of molecular function (Fig. 1D). KEGG analysis demonstrated that characteristic genes were closely associated with “PI3K − Akt signaling pathway” (*p* = 1.05 × 10^− 13^), “proteoglycans in cancer” (*p* = 5.10 × 10^− 13^), “platelet activation” (*p* = 2.12 × 10^− 15^), “chemokine signaling pathway” (*p* = 8.49 × 10^− 13^), “human T-cell leukemia virus 1 infection” (*p* = 3.05 × 10^− 14^), and “complement and coagulation cascades” (*p* = 1.55 × 10^− 15^) (Fig. 1E). Additionally, the GSEA analysis indicated that the characteristic genes were strongly correlated with “G2M checkpoint” (*p* = 3.33 × 10^− 10^), “E2F targets” (*p* = 1.00 × 10^− 10^), “epithelial mesenchymal transition” (*p* = 9.98 × 10^− 7^), “kras signaling up/DN” (*p* = 1.25 × 10^− 4^/ 1.30 × 10^− 4^), “glycolysis” (*p* = 1.74 × 10^− 3^), “interferon gamma response” (*p* = 1.13 × 10^− 3^), “inflammatory response” (*p* = 2.95 × 10^− 3^), “IL6 JAK STAT3 signaling” (*p* = 5.08 × 10^− 3^), “T signaling via NF-κB” (*p* = 7.10 × 10^− 3^), “IL2 STAT5 signaling” (*p* = 01.49 × 10^− 2^), and “interferon alpha response” (*p* = 1.30 × 10^− 2^) (Fig. 1F).

### Platelet-related prognostic risk model was constructed and validated in glioma

**Fig. 2:**
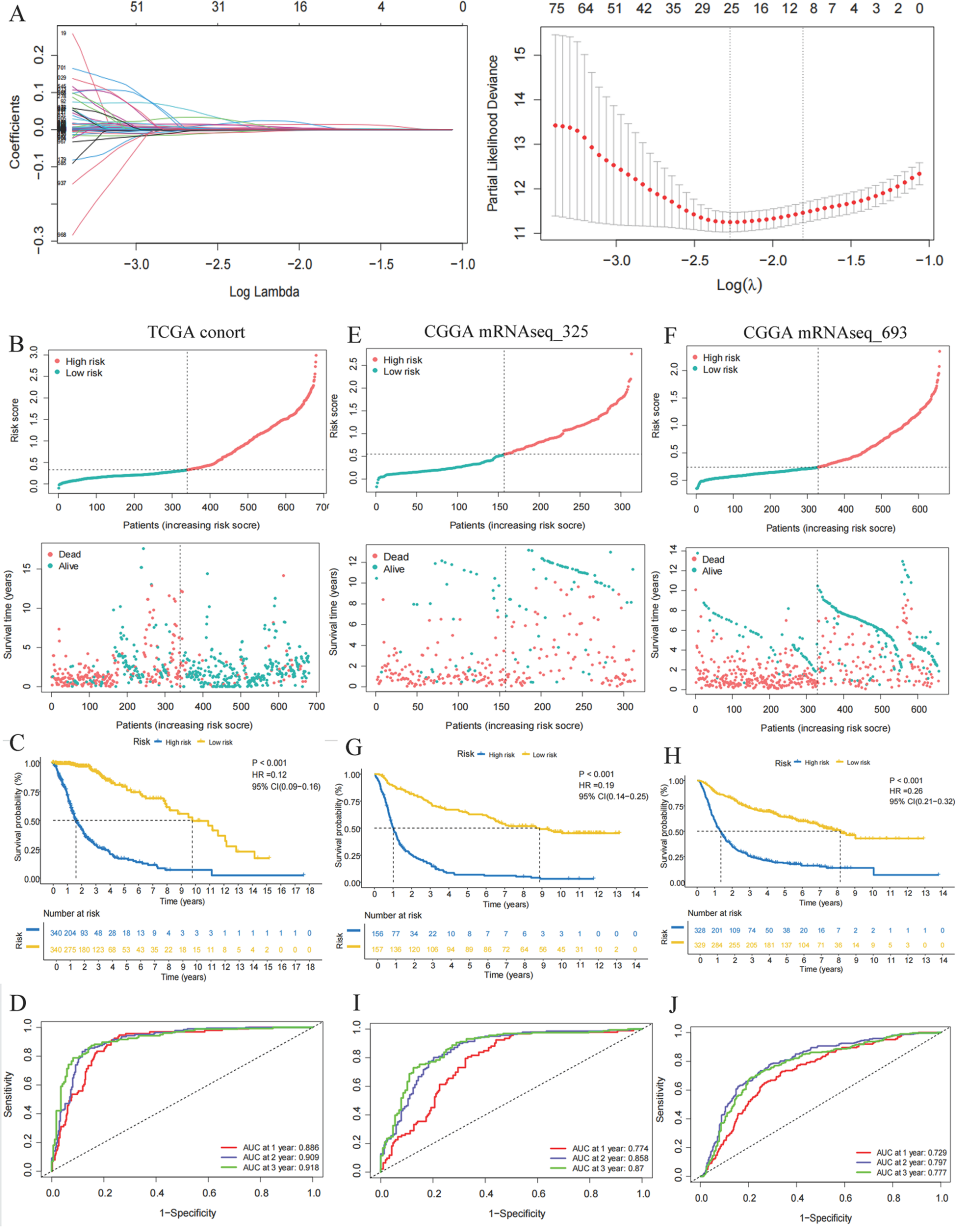
Prognostic model establishment and validation. (**A**) LASSO regression analysis. (**B**) Description of risk score and survival status by dividing glioma samples into high- and low-risk groups in TCGA training set. (**C**) KM curve describing the survival rate of glioma patients in the high- and low-risk groups in TCGA training set. (**D**) ROC curve of the predictive ability of the risk score for 1-, 2-, and 3-year survival rates in TCGA cohort. Description of risk score and survival status by dividing the samples into high- and low-risk groups in the CGGA mRNAseq_325 (**E**) and CGGA mRNAseq_693 (**F**) validation set. KM curve describing the survival rate of glioma patients in the high- and low-risk groups in the CGGA mRNAseq_325 (**G**) and CGGA mRNAseq_693 (**H**) validation sets. ROC of the predictive ability of the risk score for 1-, 2-, and 3-year survival rates in the CGGA mRNAseq_325 (**I**) and CGGA mRNAseq_693 (**J**) validation sets. TCGA, The Cancer Genome Atlas; CGGA, Chinese Glioma Genome Atlas; ROC, receiver operating characteristic; AUC, area under the curve; KM, Kaplan–Meier;

Based on the aforementioned 1030 characteristic genes, LASSO regression analysis was performed to screen the optimal gene combination for the construction of the most valuable risk model. The results revealed nine optimal platelet-related prognostic signature genes in the TCGA cohort (*p* < 0.05), including capping actin protein, gelsolin-like (*CAPG*), chloride intracellular channel 1 (*CLIC1*), galactosidase beta 1 (*GLB1*), *G* protein subunit gamma 12 (*GNG12*), kinesin family member 20 A (*KIF20A*), protein disulfide isomerase family A member 4 (*PDIA4*), sulfatase 2 (*SULF2*), t2 (*TAGLN2*), and wee1-like protein kinase (*WEE1*) (Fig. 2A). Subsequently, the risk score of samples in the TCGA cohort was derived according to the following formula: *risk score* = (1.2 × 10^− 4^ × *CAPG*) + (2.12 × 10^− 3^ × *CLIC1*) + (1.42 × 10^− 2^ × *GLB1*) + (2.36 × 10^− 3^ × *GNG12*) + (5.36 × 10^− 3^ × *KIF20A*) + (1.66 × 10^− 3^ × *PDIA4*) + (-1.16 × 10^− 3^ × *SULF2*) + (8.1 × 10^− 4^ × *TAGLN2*) +(3.40 × 10^− 3^ × *WEE1*). In addition, IHC analysis showed that the protein expression of the nine optimal signature genes between the normal and glioma tissues. It was found that *CAPG*, *CLIC1*, *GLB1*, *GNG12*, *KIF20A*, *PDIA4*, *SULF2*, *TAGLN2*, and *WEE1* were not detected in the normal tissues; while were expressed in the glioma tissues (low or medium expression) except for *GNG12* and *TAGLN2* (Supplementary Fig. 2).

In the TCGA cohort, the glioma samples were divided into high- and low-risk groups according to the median value of the risk score; as well as the samples with the risk score value below the median were used as the low-risk group (the glioma with better prognosis), while the samples with the risk score value higher the median were used as the high-risk group (the glioma with worse prognosis) (Fig. 2B). Furthermore, patients with high risk had a higher probability of death earlier than those with low risk (Fig. 2B). Regarding prognosis of glioma, the KM survival curve demonstrated that the high-risk group had a significantly lower survival rate than the low-risk group (*p* < 0.001, HR = 0.12, 95% CI = 0.09–0.16; Fig. 2C). ROC curves were generated to estimate the efficiency of the prognostic model. The areas under the curve (AUCs) for 1-, 2-, and 3-year survival were 0.886, 0.909, and 0.918, respectively, indicating that the prognostic model had a good accuracy (Fig. 2D).

In addition, the constructed prognostic model was further verified using the datasets of CGGA mRNAseq_325 and CGGA mRNAseq_693. Based on their corresponding median values of risk scores, the patient samples with glioma in the CGGA mRNAseq_325 (Fig. 2E) and CGGA mRNAseq_693 (Fig. 2F) were categorized into high- and low-risk groups. As shown in Fig. 2E, and 2F, the patients with low risk score had a lower probability of death than those with high risk. Consistently, the KM survival curve in the two validation datasets revealed that patients in the high-risk group had a poor prognosis (*p* < 0.001, HR = 0.19, 95% CI = 0.14–0.25 for CGGA mRNAseq_325; *p* < 0.001, HR = 0.26, 95% CI = 0.21–0.32 for CGGA mRNAseq_693; Fig. 2G and H). The AUCs for 1-, 2-, 3-year survival were 0.774, 0.858, and 0.87, respectively, in the CGGA mRNAseq_325 dataset (Fig. 2I). Meanwhile, the AUCs for 1-, 2-, 3-year survival were 0.729, 0.797, and 0.777, respectively, in the CGGA mRNAseq_693 dataset (Fig. 2J).

To evaluate the predictive effectiveness of the model, polygenic risk score (PRS) was subjected to an ROC analysis. The AUCs for PRS were 0.839, 0.805, and 0.761 in TCGA cohort and the CGGA mRNAseq_325 and CGGA mRNAseq_693 datasets, respectively, indicating a great predictive ability (Supplementary Fig. 3A, 3B, 3 C). To appraise the forecasting ability of the risk scores and other clinical characteristics, ROC curves for both the training and validation sets were constructed. The AUCs for risk score, gender, and age were 0.878, 0.524, and 0.810, respectively, in TCGA cohort, indicating better prognostic accuracy of the risk scores than that of other clinical features (Supplementary Fig. 3D). Similar results were obtained for the two validation sets (Supplementary Fig. 2E, 2 F). Thus, the prognostic model showed favorable efficiency in predicting the prognosis of gliomas.

### Screening of independent prognostic factor and nomogram construction

**Fig. 3:**
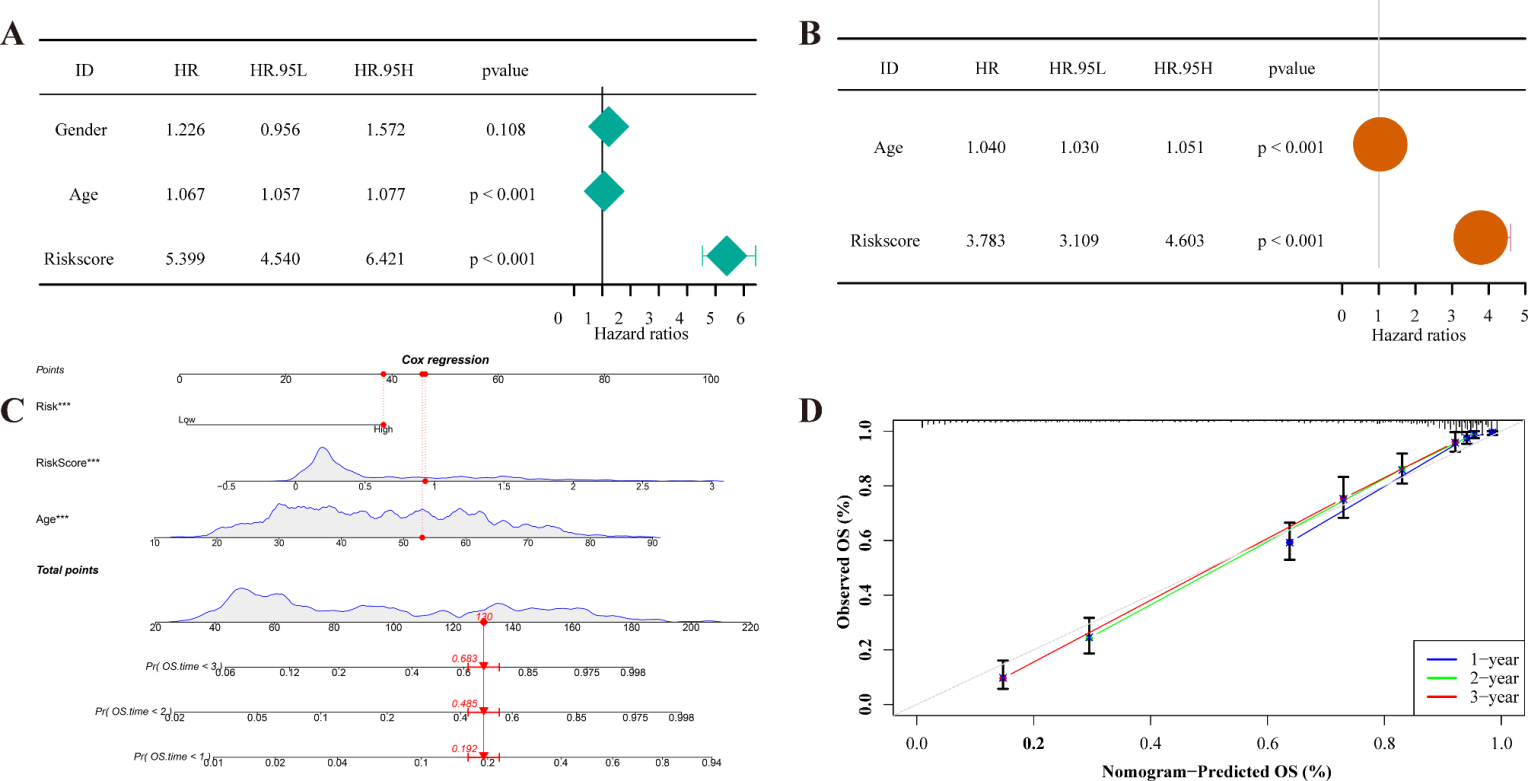
Nomogram assessing prediction efficiency of the prognostic model in TCGA cohort in glioma patients. (**A**) Univariate Cox analysis of risk scores and clinical characteristics. (**B**) Multivariate Cox analysis of risk scores and clinical characteristics. (**C**) A nomogram built based on age and risk scores. (**D**) Columnar line plot calibration curve for 1-, 2- and 3-year survival probabilities. The dashed 45° line on the calibration chart indicates the ideal prediction, whereas the X and Y axes on the chart indicate the progress and observations of the nomogram prediction, respectively. TCGA, The Cancer Genome Atlas

Univariate and multivariate Cox regression analyses were performed to investigate whether the risk score could be used as an independent predictive model for patients with glioma. Univariate Cox regression showed a statistical correlation between the risk score, age, and survival outcomes in patients with glioma (*p* < 0.001, Fig. 3A). Further, it was found that risk score and age were independent prognostic predictors of glioma after multivariate Cox analysis (*p* < 0.001, Fig. 3B). Subsequently, in the TCGA cohort, a nomogram was constructed to evaluate the relationship between each variable (age and risk score) in the prognostic model (Fig. 3C). For example, a patient aged 53 years old and with the risk score of 0.93 had about a total point of 130, with a predicted (Pr) overall survival (OS.) time < 3 of 0.683, a Pr OS. time < 2 of 0.485, and a Pr OS. time < 1 of 0.192 (Fig. 3C). Then, the performance of the nomogram was assessed using a calibration diagram. The diagram revealed a linear relationship between the nomogram-predicted prognostic model and 1-, 2-, and 3-year survival rates, indicating that the model had a high predictive effect (Fig. 3D). In summary, the risk score could be considered a credible prognostic marker for patients with gliomas.

### TME and the treatment of glioma were widely related

**Fig. 4:**
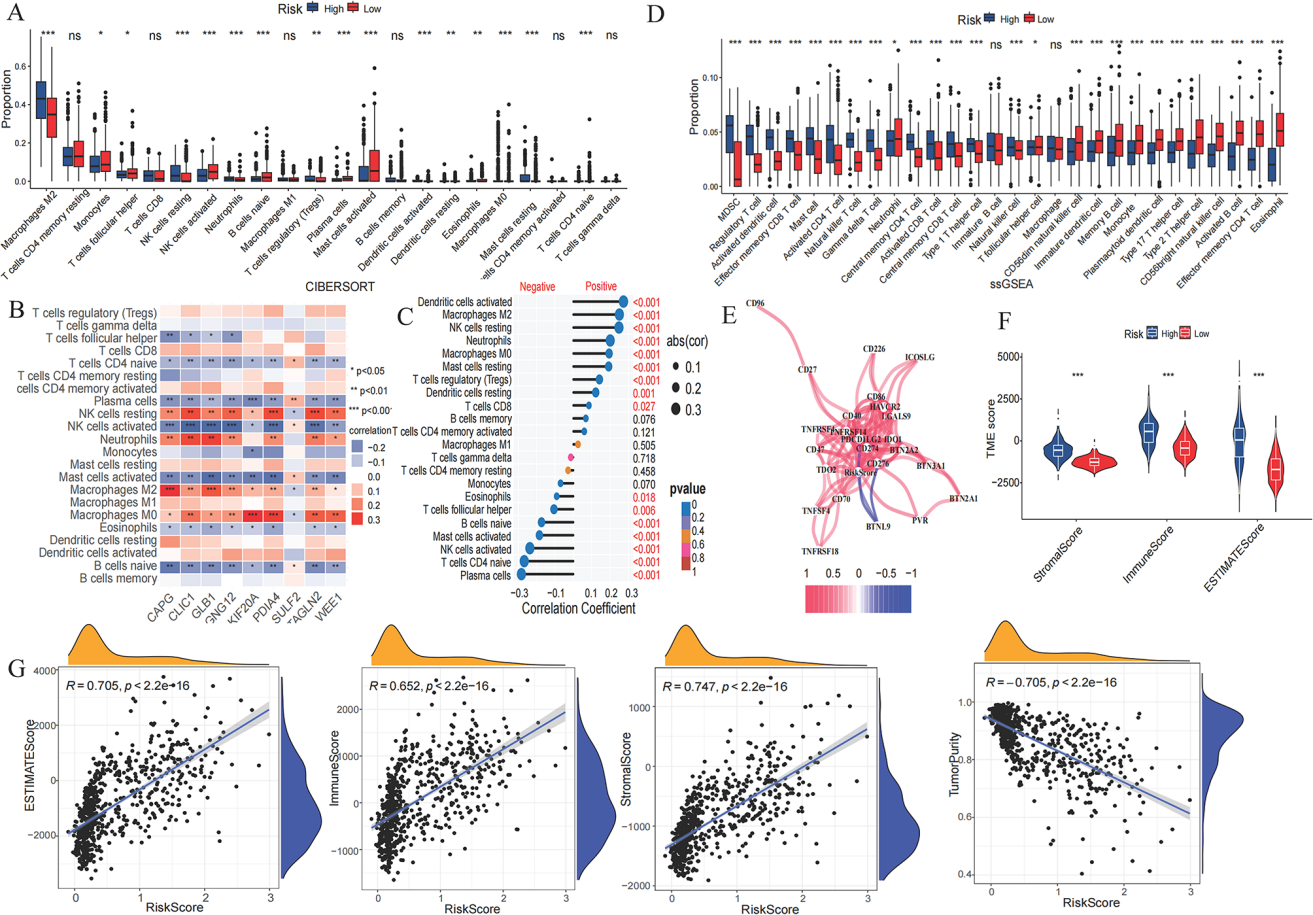
Tumor immune microenvironment between high- and low-risk groups in TCGA cohort. (**A**) CIBERSORT scores for 22 immune cells. (**B**) Correlation matrix between immunocytes and prognostic signature genes. (**C**) Correlation lollipop plot between immune cells and risk score. (**D**) ssGSEA scores for 28 immune cells. (**E**) Correlation network diagram between the risk scores and immune checkpoints. (**F**) Violin Plot depicting the TME scores of high- and low-risk group patients (**G**) Correlation analysis between the risk scores and ESTIMATE score, immune score, stromal score, and tumor purity. TCGA, The Cancer Genome Atlas; TME, tumor microenvironment; GSEA, Gene set enrichment analysis

The association between immune and the aforementioned nine optimal risk signatures was also explored in the TCGA cohort. The results of CIBERSORT revealed that most immune cells, such as Tregs, resting NK cells, and M2 macrophages, showed a higher proportion of infiltration in the high-risk group, whereas other immune cells, including naive B cells, activated NK cells, and mast cells, displayed a higher proportion of infiltration in the low-risk group (Fig. 4A). We also observed an association between prognostic signature genes and immune cells. Naive T cells CD4, activated NK cells, plasma cells, and mast cells, were negatively correlated with the expression of *CAPG*,* CLIC1*,* GLB1*,* GNG12*,* PDIA4*,* SULF2*,* TAGLN2*, and *WEE1*, while was positively correlated with *KIF20A* expression. Conversely, M2 and M0 macrophages were positively correlated with the expression of *CAPG*,* CLIC1*,* GLB1*,* GNG12*,* PDIA4*,* SULF2*,* TAGLN2*, and *WEE1*; whereas was negatively correlated with *KIF20A* expression (Fig. 4B). Aligning with the above mentioned results, plasma cells, naive T cells CD4, and activated NK cells had a negative correlation with the risk score, whereas M2 and M0 macrophages had a positive correlation with the risk score (Fig. 4C).

Furthermore, the infiltration proportions of 28 immune cells in the distinct risk groups were determined using ssGSEA. These results revealed significant differences in the proportions of the 26 immune cell types except for immature B cell (*p* > 0.05) and macrophage (*p* > 0.05), consistent with the CIBERSORT results (Fig. 4D). Afterwards, the correlation analysis of immune checkpoints and prognostic signature genes showed that the immune checkpoint of *BTNL9* was negatively correlated with the prognostic signature genes (risk score), whereas the other immune checkpoints were positively associated with the prognostic signature genes (Fig. 4E).

The TME was highly correlated with glioma prognosis and treatment outcomes, so we further analyzed the stromal, immune, and ESTIMATE scores in the high- and low-risk groups. It was found that compared with the low-risk group, the stromal, immune, and ESTIMATE scores in the high-risk group were significantly higher (*p* < 0.001, Fig. 4F). As shown in Fig. 4G, the risk score was positively correlated with stromal (*R* = 0.747, *p* < 2.2e-16), immune (*R* = 0.652, *p* < 2.2e-16), and ESTIMATE (*R* = 0.705, *p* < 2.2e-16) scores; whereas was negatively correlated with tumor purity (*R*= -0.705, *p* < 2.2e-16).

### scRNA data analysis to verify the function of prognostic signature genes

**Fig. 5:**
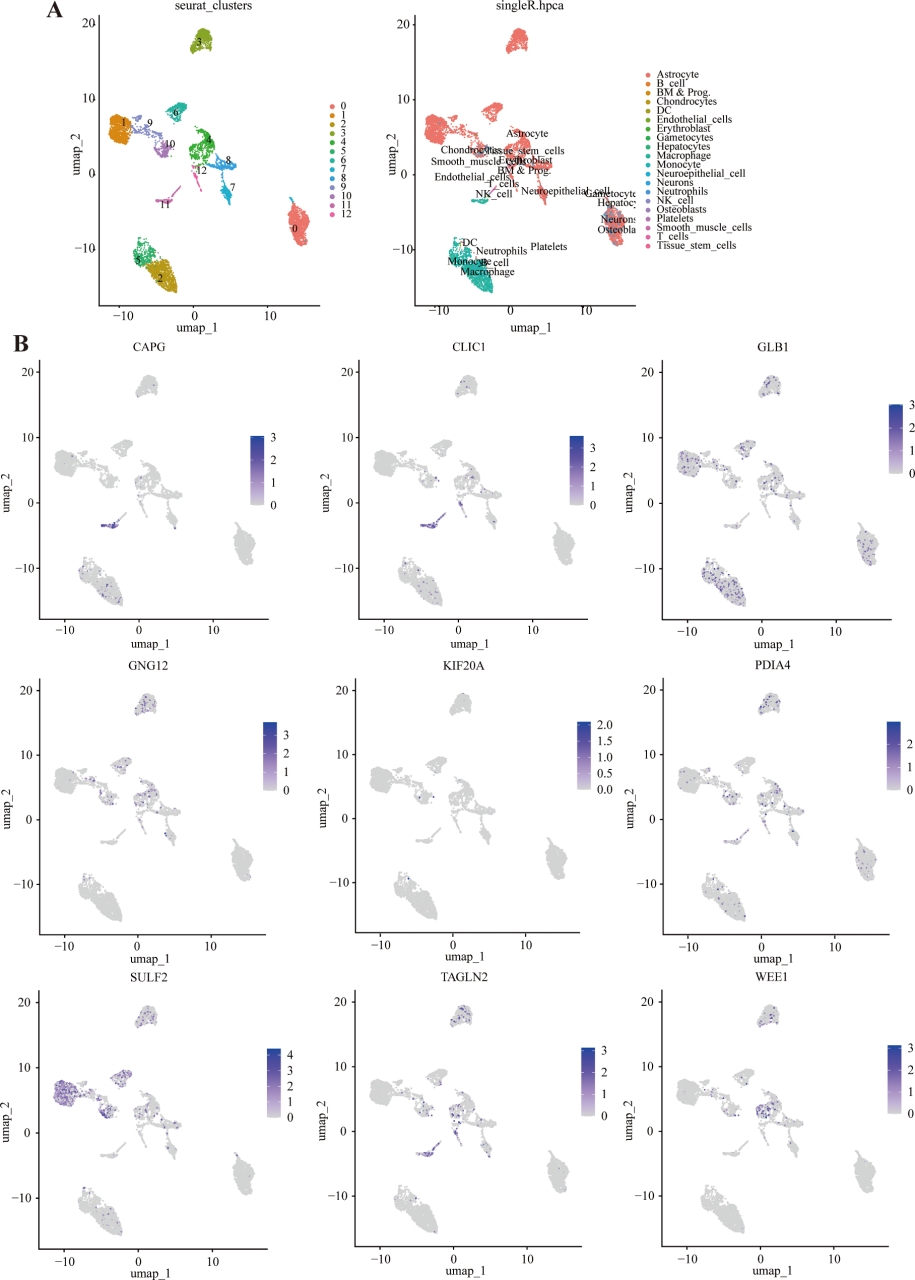
scRNA analysis of glioma. (**A**) Annotation of cell types. (**B**) The expression distributions of *CAPG*, *CLIC1*, *GLB1*, *GNG12*, *KIF20A*, *PDIA4*, *SULF2*, *TAGLN2*, and *WEE1* across different cell types. scRNA-seq, single-cell RNA sequencing; CAPG, capping actin protein, gelsolin-like; CLIC1, chloride intracellular channel 1; GLB1, galactosidase beta 1; GNG12, G protein subunit gamma 12; KIF20A, kinesin family member 20 A; PDIA4, protein disulfide isomerase family A member 4; SULF2, sulfatase 2; TAGLN2, transgelin 2

From the cellular level, the scRNA data were employed to confirm the function of the nine prognostic signature genes in glioma. Eventually, 13 cell clusters (cluster 0, 1, 2, 3, 4, 5, 6, 7, 8, 9, 10, 11 and 12) were obtained, which annotated into 20 cell types, such as astrocyte, B cell, chondrocytes, dendritic cells, endothelial cells, erythroblast, gametocytes, hepatocytes, macrophage, monocyte, neuroepithelial cell, neurons, neutrophils, platelets, T cells, and natural killer (NK) cells (Fig. 5A). Then, the expression distributions of *CAPG*, *CLIC1*, *GLB1*, *GNG12*, *KIF20A*, *PDIA4*, *SULF2*, *TAGLN2*, and *WEE1* across different cell types were analyzed. It was shown that *CAPG*,* CLIC1*, and *TAGLN2* were highly expressed in NK cells and T cells (Fig. 5B). *GLB1* was mainly expressed in epithelial cells, tissue stem cells, and macrophages; and *SULF2* were highly expressed in the astrocytes and tissue stem cells (Fig. 5B). The resting signature genes (*GNG12*,* KIF20A*,* PDIA4*, and *WEE1*) were primarily expressed in astrocytes (Fig. 5B).

### Genetic mutation, drug sensitivity speculation, and identification of potential therapeutic drugs

**Fig. 6:**
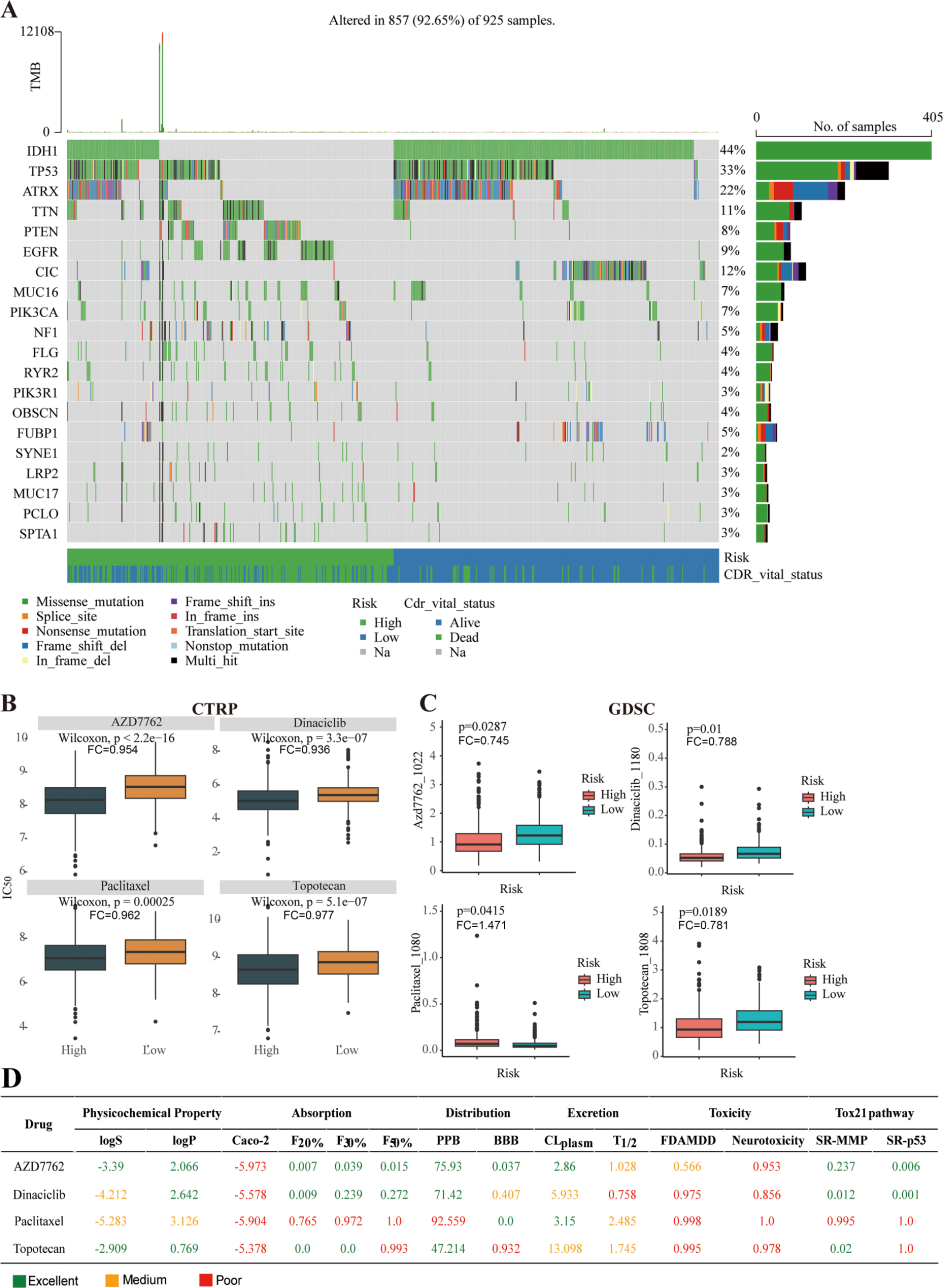
Tumor mutation and drug sensitivity analyses of patients at different risk statuses, and identification of potential therapeutic drugs for patients. (**A**) Waterfall plots of the genetic mutation features. (**B**) Sensitivity prediction of chemotherapeutic regents in the CTRP dataset. (**C**) Sensitivity prediction of chemotherapeutic regents in the GDSC dataset. (**D**) ADMET attributes of potential therapeutic drugs. CTRP, Cancer Therapeutics Response Portal; GDSC, Genomics of Drug Sensitivity in Cance

Further, gene mutation in glioma was analyzed using waterfall maps, and the top 20 mutated genes were identified and visualized as high- and low-risk groups (Fig. 6A). Among them, the main type of mutation was missense mutations. *IDH1* (44%) in glioma was the most frequently mutated gene, followed by *TP53* (33%) and *ATRX* (22%). Notably, *IDH1* mutations occurred in almost all samples in the low-risk group (Fig. 6A).

Based on the CTRP- and GDSC-derived drug response data, drug sensitivity was evaluated in the high- and low- risk groups. Prospective drugs with high sensitivity in high-risk populations were also explored. In total, 42 drug candidates or compounds were identified from the CTRP and GDSC databases. Among them, four drugs or compounds (AZD7762, dinaciclib, paclitaxel, and topotecan) were observably diverse in the two risk groups. Based on the CTRP database, the IC_50_ values of AZD7762 (FC = 0.954), dinaciclib (FC = 0.936), paclitaxel (FC = 0.962), and topotecan (FC = 0.977) were significantly lower in the high-risk group than those in the low-risk group (*p* < 0.05, Fig. 6B). According to the GDSC database, the trend of IC_50_ values of AZD7762 (FC = 0.954), dinaciclib (FC = 0.936), and topotecan in the two risk groups was similar with that based on the CTRP database (Fig. 6C). However, in the GDSC database, the IC_50_ value of paclitaxel (FC = 1.471) in the high-risk group was evidently higher than that in the low-risk group (*p* < 0.05, Fig. 6C).

In addition, to further investigate the superiority of drug candidates for all glioma patients, ADMET analysis was performed. We found that AZD7762 was the primary drug with superior absorption, distribution, metabolism, excretion, and toxicity than other compounds, followed by dinaciclib, topotecan and paclitaxel (Fig. 6D).

## Discussion

Gliomas are malignant brain tumors that seriously threaten human safety [43], representing 81% of all central nervous system (CNS) cancers [6]. Platelets are closely correlated with the occurrence, metastasis, and recurrence of gliomas, which may significantly affect patient prognosis [19]. Although glioma treatments have progressed in recent years [6, 44, 45], the prognosis remains unsatisfactory. Therefore, screening for novel platelet-related prognostic biomarkers is crucial. In this study, we identified 1,030 characteristic genes; nine platelet-related prognostic genes (*CAPG*,* CLIC1*,* GLB1*,* GNG12*,* KIF20A*,* PDIA4*,* SULF2*,* TAGLN2*, and *WEE1*) were identified based on TCGA cohort, and their expression and function were verified. The glioma samples were categorized into high- and low-risk groups according to the risk score, and the constructed prognostic model showed good performance in both the training and validation datasets. Moreover, a nomogram model constructed using the risk scores and age revealed that the risk score could be a reliable prognostic signature for patients with glioma. We also found that the prognostic signature genes and models were highly correlated with immune cells in gliomas. Finally, the analysis of sensitivity to chemotherapy drugs demonstrated AZD7762 as a potential effective drug for glioma treatment.

Glioma development is complex, and the factors involved are not entirely clear, resulting in challenging clinical treatment and poor prognoses [46]. Finding reliable biomarkers to predict the disease is a major challenge in personalizing treatment and improving outcomes for patients with glioma [47, 48]. Previous studies reported that the platelet-to-lymphocyte ratio and platelet count are closely related to the development of glioma [23, 24], suggesting that PRGs might have prognostic value in glioma. In this study, 1,030 DEPRGs were identified, indicating that PRGs vary widely in gliomas, which is consistent with reports that PRGs differ in different types of cancers [49, 50]. Enrichment analysis revealed that DEPRGs were largely enriched in pathways such as platelet activation, epithelial-mesenchymal transition, and glycolysis. This study suggested that PRGs may influence the development and prognosis of glioma by regulating glucose metabolism in vivo and the epithelial-mesenchymal transition pathway.

Platelets are known to influence glioma occurrence, and in this study, we identified nine platelet-related prognostic signature genes—*CAPG*,* CLIC1*,* GLB1*,* GNG12*,* KIF20A*,* PDIA4*,* SULF2*,* TAGLN2*, and *WEE1*. The IHC analysis showed that in addition to the expression of *GNG12* and *TAGLN2*, the expression of *CAPG*, *CLIC1*, *GLB1*, *KIF20A*, *PDIA4*, *SULF2*, and *WEE1* were all expressed in the glioma tissues than in the normal tissues (low or high). Relationships between these genes and cancer have also been reported. The expression level of CAPG is higher in glioma tissues than in normal tissues [51], which is in line with our results. The expression of CLIC1 mRNA and protein is significantly elevated in high-grade gliomas and is enhanced with the increase in tumor WHO grade [52]. SULF2 is the best tumor-dependent protein for glioma because the extracellular sulfatase SULF2 activates the RTK pathway [53]. PDIA4, a member of the protein disulfide isomerase family, promotes apoptosis by influencing aerobic glycolysis of metabolites, thus inhibiting GBM proliferation [54]. KIF20A deficiency can suppress the proliferation and migration of glioma cell [55]. GNG12 belongs to the G protein family, and GNG12-AS1 stimulates or suppresses the proliferation and relocation of glioma cells by influencing the KT/GSK-3β/β-catenin pathway activity [56]. TAGLN2 overexpression, an actin-binding protein, significantly promotes the proliferation and relocation of glioma cells [57]. WEE1 is a protein kinase whose expression increases with the increase of malignant degree of glioma [58]. However, increased GLB1 levels lead to increased survival without prostate-specific antigen (PSA) in prostate cancer [59], which is contradictory with our outcomes, as well as the specific roles of GLB1 in glioma need to be further explored. These reports, together with our findings, it can be inferred that all prognostic signature genes (except for *GNG12* and *TAGLN2*) were poor prognostic factors for glioma, and were highly expressed in glioma tissues [56, 58–65]; but the detailed actions of these genes in gliomas warranted to be unearthed using in vitro and in vivo experiments. Consequently, the platelet-related signature genes (*CAPG*, *CLIC1*, *GLB1*, *GNG12*, *KIF20A*, *PDIA4*, *SULF2*, *TAGLN2*, and *WEE1*) are closely associated with the prognosis of patients with glioma; as well as the proposed prognostic risk model based on these platelet-related signature genes may be an effective tool for predicting the prognosis of gliomas.

Gliomas recruit immune regulatory cells, which, together with macrophage-related tumor-associated cells, promote immunosuppressive functions in the tumor immune microenvironment [66]. Thus, the immune system determines the development and effectiveness of tumor treatment. Platelets are strongly associated with gliomas and the immune microenvironment and may promote tumor development by affecting the TME [67]. The interaction between tumors and platelets activates platelets, which generate normal MHC class I to coat tumor cells and help them evade immune surveillance [68]. In addition, activated platelets release transforming growth factor beta (TGF-β1) into the TME to promote the spread of tumor cells to other organs [67]. Activated platelets can release the CD40 ligand, inhibiting the migration ability of CD4CD25Foxp3 regulatory T cells and eventually affecting the antitumor immunity of gliomas [20]. In this study, most immune cells, including B cells, T cells CD8 and Tregs, differed in the high- and low-risk groups and had different degrees of correlation with prognostic signature genes in gliomas. Tregs could inhibit tumor growth [69], and ADAM10 released by glioma cells can induce regulatory B cells, inhibit the activity of CD8 + T cells, and induce Tregs [70]. Neoadjuvant immune checkpoints can guide targeted therapy for GBM; for instance, Siglec-9, an immune checkpoint molecule on macrophages, can directly activate CD4 T and CD8 T cells to affect the treatment of GBM [71]. In this study, the immune checkpoints and prognostic signature genes were significantly correlated. Therefore, targeted regulation of prognostic signature genes is conducive to inhibit or promote platelets to affect angiogenesis and other processes of tumor cells, thereby affecting the immune function of the TME. This finding is consistent with the conclusions of a recent research [18].

PRGs are mainly expressed in platelets, and play a key role in hemostasis, clotting, and the immune system. From the cellular level, we found that the identified signature genes were primarily expressed in astrocytes, NK cells, and macrophage. After central nervous system injury, astrocytes are reactively activated, which are the key cells involved in the repair mechanism after injury. Reactive astrocytes are an integral part of the glioma microenvironment [72]. Lin et al. [73] reported that astrocytes could protect glioma cells from chemotherapy, and up-regulate survival genes through gap junction communication. NK cells, as effector lymphocytes of the innate immune system, have a wide range of tumor recognition and killing mechanisms, and are an important part of the first line of defense against malignant cells. A previous investigation identified that NK cells-associated genetic signatures could predict glioma malignancy and patient survival [74]. Macrophages are heterogeneous, as well as their phenotype and function are regulated by the surrounding microenvironment. Macrophages are typically found in two distinct subpopulations: M1 macrophages, which has proinflammatory effects and are polarized by Th1; and M2 macrophages, which have anti-inflammatory and immunomodulatory effects, and are polarized by Th2 [75]. Pyonteck et al. [76] demonstrated that CSF-1R inhibition could alter the polarization of macrophages, and suppress the development of glioma. Taken together, we can speculate that the identified signature genes expressed in astrocytes, NK cells, and macrophage may be mainly related to immunity and inflammation; as well as astrocytes, NK cells, and macrophage may participate in the occurrence and progression of glioma.

Platelets protect tumor cells from the cytotoxicity of chemotherapy drugs [77]. Activated platelets release molecules and growth factors such as EGF, PDGF, TGF-β, IGF and CCL5 to promote the expansion of ovarian cancer cells, protecting tumor cells from chemotherapy [78]. Platelets also impair the adaptive immune response against tumors in the TME. Therefore, inhibiting platelet activity can improve the efficacy of immunotherapy [77]. In this study, active components of platelet-related prognostic target proteins were screened and identified. Moreover, we found that AZD7762 could bind to platelet-related prognostic signature target proteins, highlighting its potential for glioma treatment. Temozolomide (TMZ), a commonly used drug for glioma treatment, and a combination of TMZ and AZD7762 can induce synergistic cytotoxic effects in human glioma cells [79]. In addition, AZD7762 lowered the metabolism of pembrolizumab and reduced tumor size in these models by synergizing with gemcitabine [80]. Consequently, the synergy of AZD7762 with other drugs can reduce tumor growth, which has value in guiding clinical medication and prognosis of glioma.

This study had some limitations. While the establishment of the prognostic signature genes in study was validated using datasets, but a large amount of clinical data from patients with glioma is still needed to support our findings. Additionally, the mechanism underlying the immune response of prognostic signature genes in gliomas requires further investigation. Finally, the effects of the selected drug candidates on glioma prognosis requires further verification.

## Conclusion

Nine platelet-related prognostic genes (*CAPG*,* CLIC1*,* GLB1*,* GNG12*,* KIF20A*,* PDIA4*,* SULF2*,* TAGLN2*, and *WEE1*) were identified. We constructed a prognostic model and validated it using external datasets, demonstrating its accuracy in predicting its gliomas. TME analysis showed that almost all immune cells, including B cells, CD8 T cells, NK cells, and Tregs, differed in the high- and low-risk groups and had different degrees of correlation with prognostic signature genes in gliomas. In addition, AZD7762 may be an appropriate candidate for the treatment of gliomas. Our study identified that platelets could influence glioma prognosis and provided a new strategy for the clinical treatment and prognosis of gliomas. Additionally, we provided a new research direction and theoretical basis for the clinical targeting of platelets and immunotherapy.

## Electronic Supplementary Material

Below is the link to the electronic supplementary material.

**Supplementary Material 1**: **Supplementary Table 1** List of 4,367 platelet-related genes in this study

**Supplementary Material 2**: **Supplementary Figure 1** Study flow chart.

**Supplementary Material 3**: **Supplementary Fig. 2** Immunohistochemical of prognostic signature proteins expression in HPA database. HPA, Human Protein Atlas.

**Supplementary Material 4**: **Additional Fig. 3**. Validation of predictive efficiency of the prognostic ris*k* model. (A) The ROC of PRS in survival monitoring in TCGA cohort. (B-C) The ROC of PRS in survival monitoring in CGGA mRNAseq_325 and CGGA mRNAseq_693 datasets. (D) The ROC of risk score, gender, and age in TCGA cohort. (E-F) The ROC of risk score, gender, and age in CGGA mRNAseq_325 and CGGA mRNAseq_693 datasets. ROC, receiver operating characteristic; PRS, polygenic risk score; CGA, The Cancer Genome Atlas; CGGA, Chinese Glioma Genome Atlas

## Data Availability

No datasets were generated or analysed during the current study.

## Abbreviations

GBM: Glioblastoma
CAPG: Capping actin protein, gelsolin-like
CLIC1: Chloride intracellular channel 1
GLB1: Galactosidase beta 1
GNG12: G protein subunit gamma 12
KIF20A: Kinesin family member 20 A
PDIA4: Protein disulfide isomerase family A member 4
SULF2: Sulfatase 2
TAGLN2: Transgelin 2
NK: Natural killer
Tregs: Regulatory T cells
TME: Tumor microenvironment
TCGA: The Cancer Genome Atlas
CGGA: Chinese Glioma Genome Atlas
scRNA-seq: Single-cell RNA sequencing
PRGs: Platelet-related genes
GSEA: Gene set enrichment analysis
GO: Gene Ontology
KEGG: Kyoto Encyclopedia of Genes and Genomes
ROC: Receiver operating characteristic
AUC: Area under the curve
CTRP: Cancer Therapeutics Response Portal
GDSC: Genomics of Drug Sensitivity in Cancer
IC50: Half-maximal inhibitory concentration
KM: Kaplan–Meier
DEGs: Differentially expressed genes
DEPRGs: Differentially expressed platelet-related genes
TGF-β1: Transforming growth factor beta
PRS: Polygenic risk score
TMZ: Temozolomide
