# Supplementary figures and images for: A novel risk model consisting of nine platelet-related gene signatures for predicting prognosis, immune features and drug sensitivity in glioma
Source: Hereditas. 2024 Dec 20;161:52. doi: 10.1186/s41065-024-00355-7 (PMC11662788; doi:10.1186/s41065-024-00355-7)

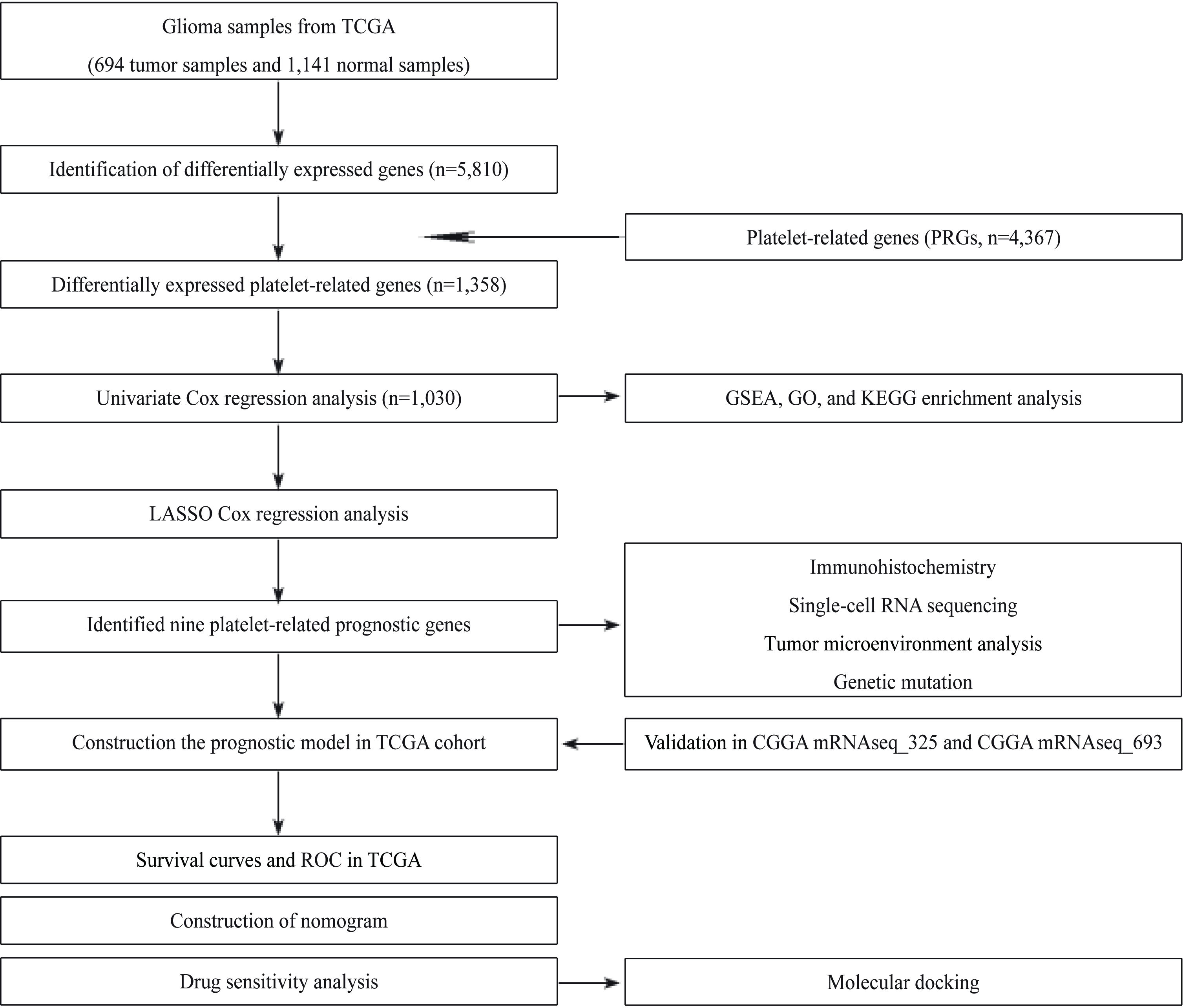

Supplement: Supplementary file 2 — Supplementary Material 2: Supplementary Figure 1 Study flow chart. [file 41065_2024_355_MOESM2_ESM.tif]

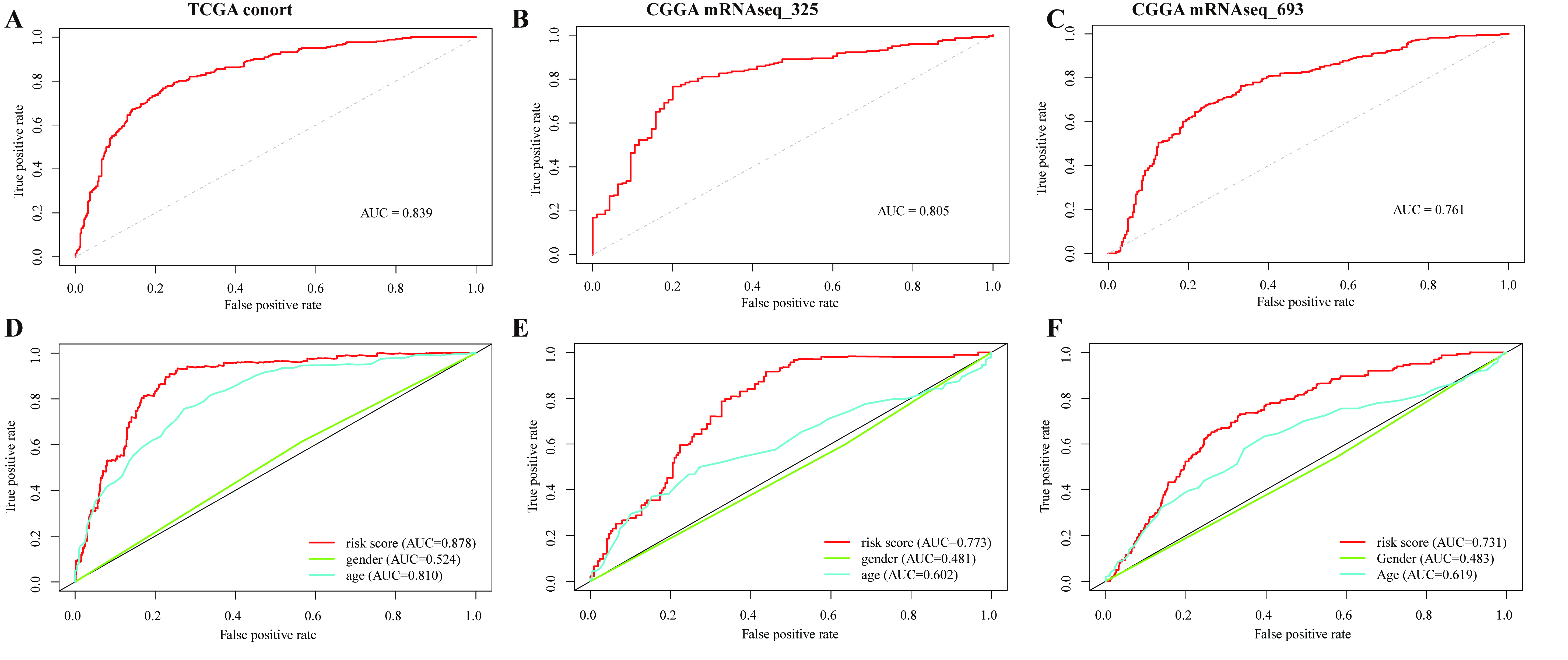

Supplement: Supplementary file 3 — Supplementary Material 3: Supplementary Fig. 2 Immunohistochemical of prognostic signature proteins expression in HPA database. HPA, Human Protein Atlas. [file 41065_2024_355_MOESM3_ESM.tif]

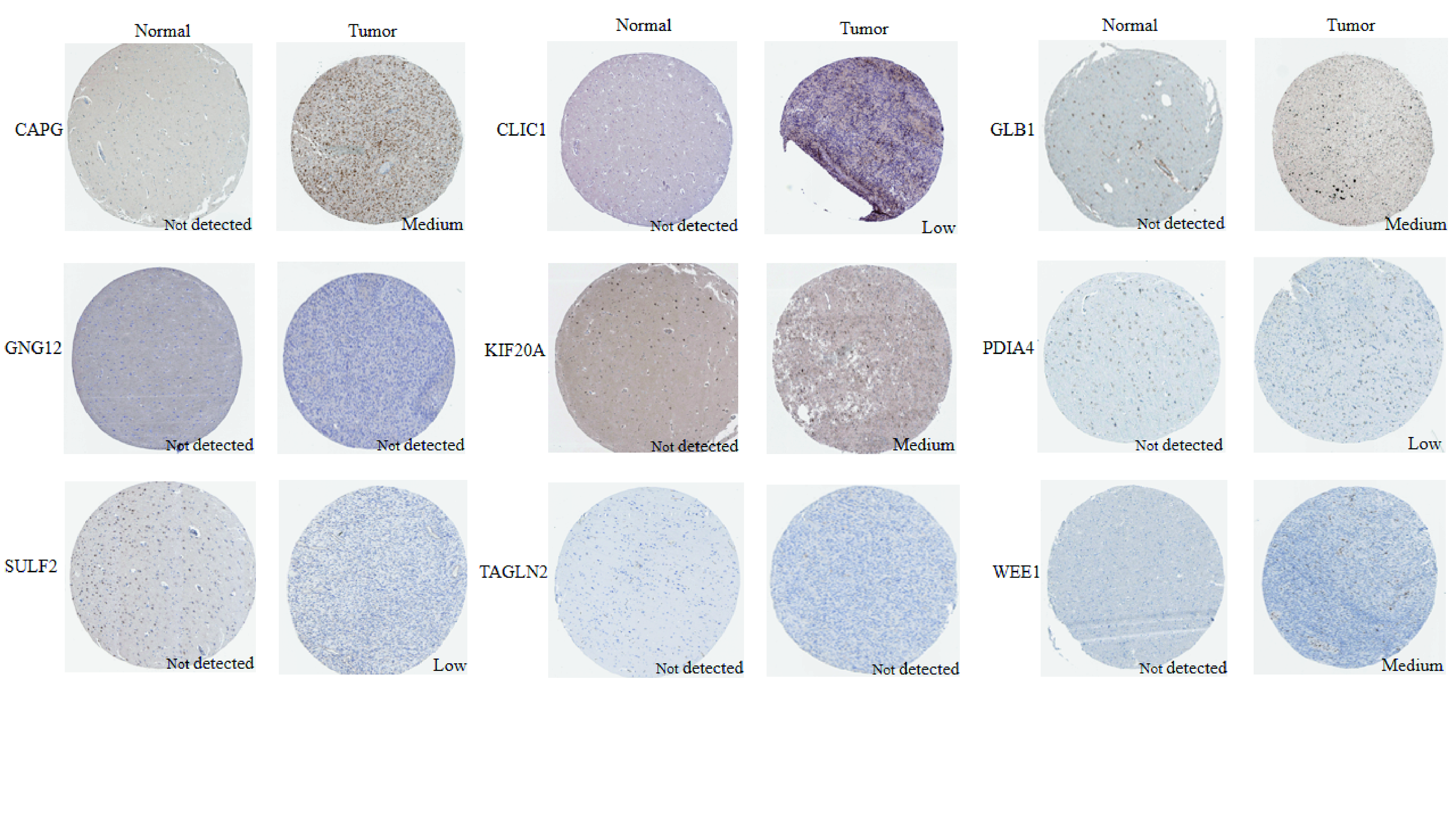

Supplement: Supplementary file 4 — Supplementary Material 4: Additional Fig. 3. Validation of predictive efficiency of the prognostic risk model. (A) The ROC of PRS in survival monitoring in TCGA cohort. (B-C) The ROC of PRS in survival monitoring in CGGA mRNAseq_325 and CGGA mRNAseq_693 datasets. (D) The ROC of risk score, gender, and age in TCGA cohort. (E-F) The ROC of risk score, gender, and age in CGGA mRNAseq_325 and CGGA mRNAseq_693 datasets. ROC, receiver operating characteristic; PRS, polygenic risk score; CGA, The Cancer Genome Atlas; CGGA, Chinese Glioma Genome Atlas [file 41065_2024_355_MOESM4_ESM.tif]
